# Sensor-Based Automatic Recognition of Construction Worker Activities Using Deep Learning Network

**DOI:** 10.3390/s25133988

**Published:** 2025-06-26

**Authors:** Ömür Tezcan, Cemil Akcay, Mahmut Sari, Muhammed Cavus

**Affiliations:** 1 Institute of Science, Istanbul University-Cerrahpaşa, 34320 Istanbul, Türkiye; omurtezcan@gmail.com; 2 Department of Architecture, Istanbul University, 34116 Istanbul, Türkiye; cakcay@istanbul.edu.tr; 3 Department of Construction, Kırşehir Ahi Evran University, 40100 Kırşehir, Türkiye; mahmutsari@ahievran.edu.tr; 4 Department of Mathematics, Physics and Electrical Engineering, Northumbria University, Newcastle Upon Tyne NE1 8SA, UK; 5 School of Engineering, İskenderun Technical University, 31200 Iskenderun, Türkiye

**Keywords:** human activity recognition, deep learning, LSTM, construction automation, motion sensors, BiLSTM, wearable sensors, productivity analysis

## Abstract

The adoption of automation technologies across various industries has significantly increased in recent years. Despite the widespread integration of robotics in many sectors, the construction industry remains predominantly reliant on manual labour. This study is motivated by the need to accurately recognise construction worker activities in labour-intensive environments, leveraging deep learning (DL) techniques to enhance operational efficiency. The primary objective is to provide a decision-support framework that mitigates productivity losses and improves time and cost efficiency through the automated detection of human activities. To this end, sensor data were collected from eleven different body locations across five construction workers, encompassing six distinct construction-related activities. Three separate recognition experiments were conducted using (i) acceleration sensor data, (ii) position sensor data, and (iii) a combined dataset comprising both acceleration and position data. Comparative analyses of the recognition performances across these modalities were undertaken. The proposed DL architecture achieved high classification accuracy by incorporating long short-term memory (LSTM) and bidirectional long-term memory (BiLSTM) layers. Notably, the model yielded accuracy rates of 98.1% and 99.6% for the acceleration-only and combined datasets, respectively. These findings underscore the efficacy of DL approaches for real-time human activity recognition in construction settings and demonstrate the potential for improving workforce management and site productivity.

## 1. Introduction

In recent years, human activity recognition (HAR) has garnered significant attention due to its wide-ranging applicability in various domains, including healthcare, smart environments, and industrial safety. A growing body of research has demonstrated the superior performance of deep learning (DL) algorithms, particularly convolutional neural networks (CNNs) and long short-term memory (LSTM) networks, in extracting meaningful temporal patterns from sensor data [1]. Comparative studies emphasise that LSTM-based models are particularly effective in modelling sequential dependencies, making them suitable for real-time activity recognition tasks using smartphone and wearable sensor data [2,3]. Moreover, integrating physiological and biological sensor data with DL approaches has been shown to enhance recognition accuracy further, underscoring the adaptability of LSTM networks across diverse sensor modalities [4]. In addition to model architecture, sensor placement has emerged as a critical factor influencing classification performance. A comprehensive analysis by [5] revealed that multi-sensor configurations, particularly combining sensors located at the wrist, waist, and ankle, contribute to improved robustness and precision in HAR systems. Similarly, the study by [6] demonstrated that LSTM models integrated with IMU sensors on embedded microcontroller platforms can achieve up to 99% classification accuracy, making them highly practical for deployment in real-world applications. Furthermore, the hybrid architecture of 3DCNN and LSTM proposed by [7] achieved superior performance in HAR tasks involving complex motion sequences. Collectively, these findings validate the methodological approach of this study, which leverages multi-position MPU-6050 IMU sensor (InvenSense Inc., San Jose, CA, USA) sensor data and a deep LSTM-based architecture to recognise construction worker activities with high precision.

The remainder of this paper is structured as follows. Section 2 reviews the relevant literature on HAR, with an emphasis on sensor-based and DL approaches. Section 3 describes the methodological framework, including the DL architecture. Section 4 details the activity selection and sensor setup. Section 5 outlines the data processing pipeline, and Section 6 presents the experimental setup and results. Section 9 concludes with implications and directions for future research.

## 2. Literature Review

### 2.1. Human Activity Recognition

HAR refers to developing automated systems that identify and classify human actions using computational technologies tailored to user needs [8]. Research in this domain has grown substantially in recent years, driven by advances in artificial intelligence (AI), machine learning (ML), and ubiquitous computing. A literature review highlights a notable surge in HAR-related studies since 2015, particularly with the increasing availability of wearable devices and sensing technologies.

Typically, HAR systems rely on various modalities for data acquisition, with sensors and camera-based imagery being the most common. Audio-based activity recognition has also been explored, though to a much more limited extent. According to Cook et al. [9] and Hussain et al. [10], HAR can be broadly categorised into two primary types: video-based and sensor-based approaches. However, Sherafat et al. [11] proposed a more granular classification, dividing HAR methods into kinematic-based, image-based, and sound-based systems. The present study adopts a sensor-based HAR methodology, which offers advantages in both accuracy and data processing efficiency.

**Figure 1:**
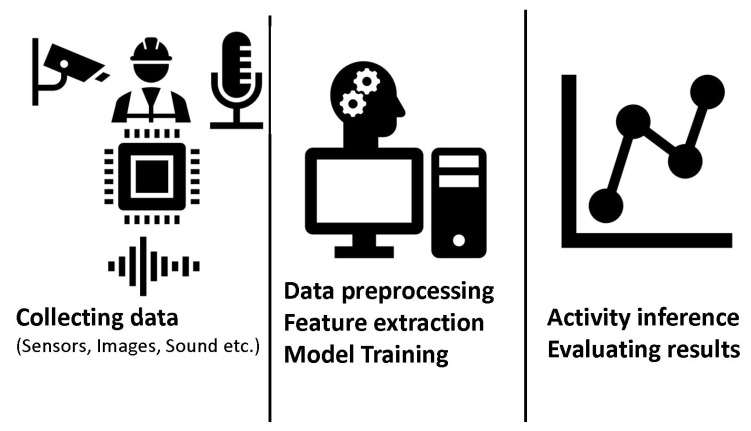
General framework for HAR.

In general, all three HAR modalities share a common three-stage workflow: (i) data acquisition, (ii) feature extraction and ML-based training, and (iii) classification through DL models (Figure 1).

Our review of existing studies indicates that researchers typically collect proprietary datasets in image- and sound-based HAR applications. In contrast, sensor- and kinematic-based approaches often utilise publicly available datasets. Although capturing audio and visual data is relatively straightforward, these modalities are highly susceptible to environmental conditions—such as lighting, noise, dust, and weather—that can significantly degrade data quality. Moreover, visual data impose considerable computational and storage requirements, which prolong data processing times compared to sensor or audio-based methods.

Chavarriaga et al. [12] categorised HAR systems by sensor placement into three distinct types: on-body sensors, in-object sensors, and environment-integrated sensors. Expanding upon this, Wang et al. [13] introduced a hybrid approach that integrates two or more sensor types to enhance recognition performance.

**Figure 2:**
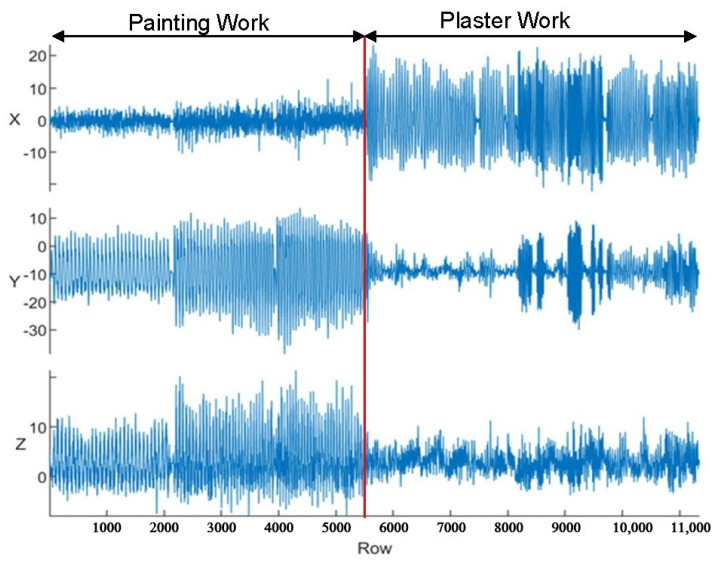
Accelerometer data across *x*-, *y*-, and *z*-axes for painting and plastering activities.

Among these, on-body sensors are the most prevalently employed in HAR research. These typically include accelerometers, gyroscopes, magnetometers, or composite inertial measurement units (IMUs). The signals obtained from these devices—such as acceleration and angular velocity—are intrinsically linked to human movement patterns. Figure 2 illustrates a representative plot of accelerometer readings collected in this study, distinguishing between three painting and three plastering workers.

### 2.2. Human Activity Recognition Studies Using Sensors

Both ML and DL approaches have been extensively applied in sensor-based HAR, particularly in studies employing on-body sensors. Among the various sensor types, accelerometers are the most widely utilised, often in combination with gyroscopes and magnetometers. These inertial sensors form the basis of many HAR systems due to their portability, low cost, and ability to capture dynamic movement data. A notable trend in the literature is the predominance of HAR studies focused on daily activity recognition, largely attributed to the widespread availability of public datasets tailored to such applications.

Wang et al. [13] and Chen et al. [14] conducted comprehensive reviews of sensor-based HAR using public datasets. Across 15 datasets—such as UCI Smartphone, Skoda Checkpoint, Pamap2, Opportunity, USC-HAD, WISDM, DSADS, Darmstadt Daily Routines, Actitracker, SHO, MHEALTH, Daphnet Gait, ActiveMiles, HASC, and Heterogeneous—a typical median of 10 subjects and 6 activity classes was observed, with a median sampling frequency of 50 Hz. The UCI HAR dataset, one of the most widely employed, consists of six labelled activities captured via a smartphone sensor worn at the waist and sampled at 50 Hz. This configuration was mirrored in the present study. However, our approach employed a more comprehensive sensor setup with 11 devices on various body parts, including the arms, legs, chest, waist, and head.

**Figure 3:**
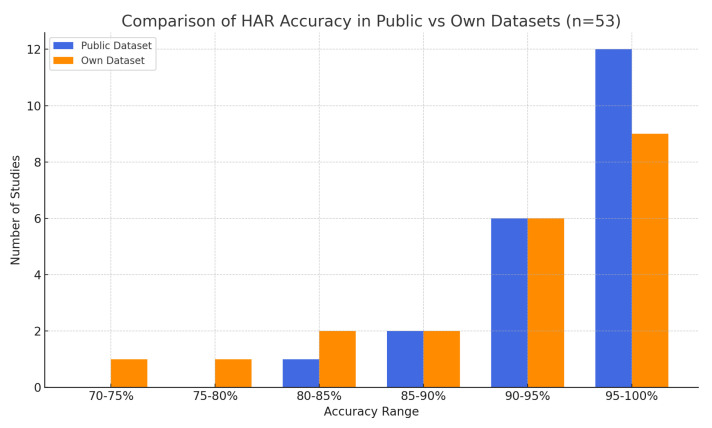
Accuracy rates of DL-based HAR studies by dataset type.

To contextualise the performance of existing DL-based HAR systems, a review of 53 prior studies was conducted. These studies were analysed based on sensor configurations, activity class complexity, dataset sources, and overall classification performance. Reported accuracies ranged from 70% to 100%, with a mean accuracy of 92.9% and a median of 94.4%. As illustrated in Figure 3, studies utilising public datasets—such as UCI HAR, WISDM, and Opportunity—tended to outperform those relying on proprietary datasets, with a mean accuracy of 94.6% compared to 91.1%. This discrepancy may be attributed to the consistency, size, and quality of public datasets, which often feature preprocessed, balanced data. The present study advances this research by demonstrating state-of-the-art accuracy (up to 99.6%) using a bespoke sensor dataset collected from real construction site activities, addressing the performance gap under complex and realistic conditions.

While DL-based HAR studies frequently employ public datasets, notable exceptions exist in the medical domain, particularly those focusing on conditions such as Parkinson’s disease. Researchers often curate their own datasets in such contexts to capture clinically relevant motion characteristics such as gait freezing and respiratory disturbances.

For instance, Cheng et al. [15] collected data from 44 individuals diagnosed with Parkinson’s disease and 35 healthy controls using smartphone sensors sampled at 20 Hz. They successfully recognised six distinct activities, including walking, using an LSTM network and achieved an accuracy of 98%. Similarly, Lee et al. [16] used smartphone-based tri-axial accelerometer data collected from five graduate students to classify walking, running, and standing still. Data were sampled at 1 Hz, with smartphones placed in different locations (e.g., hand, pocket, handbag). Using random forest and CNN models, classification accuracies of 89.1% and 92.7% were reported for CNN and random forest, respectively.

Gjoreski et al. [17] evaluated both ML and DL techniques for daily activity classification using wrist-mounted accelerometer data in another comparative study. Across two public datasets, the random forest classifier achieved an accuracy of 74.6%, while the CNN-based approach reached 75.5%. Furthermore, Chen et al. [18] employed CNNs to recognise eight different human activities using data from a three-axis accelerometer, achieving an overall classification accuracy of 93.8%.

These studies underscore the viability of sensor-based HAR, particularly when enhanced by DL architectures. However, sensor placement, sampling frequency, activity complexity, and dataset characteristics significantly influence model performance.

### 2.3. Worker Activity Recognition with Sensors in Construction

While video and audio-based activity recognition studies have been conducted within the construction sector, kinematic-based approaches remain relatively limited. Notably, most of these kinematic studies rely on classical ML algorithms, and to our knowledge, no DL-based HAR study has yet been fully realised within construction applications.

Image-based activity recognition in construction has predominantly focused on the identification of operational parameters such as soil movement, the volume of excavated or transported materials, and the detection of hazardous or unsafe worker behaviours from video feeds [19,20,21,22,23,24,25]. However, sensor-based studies offer the advantage of being less susceptible to environmental interference and do not require continuous visual line-of-sight.

One of the earliest applications of kinematic sensor-based HAR in construction was conducted by Cezar [26], who recognised four activity types—hammering, sawing, sweeping, and drilling—using data sampled at 25 Hz from smartwatches worn by three workers. A total of 46 statistical features were extracted from 1.6-s time windows, and five different ML classifiers were evaluated. The highest reported classification accuracy was 91%.

Lim et al. [27] explored the recognition of walking, slipping, and near-miss events using smartphones placed in the waist pockets of three male workers. Sensor data were sampled at 100 Hz and segmented into 2.4-s windows (240 samples). Features such as the mean, standard deviation, and peak values were extracted and fed into an artificial neural network (ANN), yielding accuracy rates of 100% for walking, 88% for slipping, and 94% for near-miss detection.

Akhavian and Behzadan [28] investigated seven construction-related activities, including sawing, hammering, turning a wrench, loading/unloading a wheelbarrow, and pushing/pulling loads. Data were collected via smartphones affixed to workers’ arms, sampled at 100 Hz. Each 1.28-s segment (128 samples) yielded 54 extracted features. Various classifiers were applied, including ANN, decision trees, K-nearest neighbours (KNNs), logistic regression (LR), and support vector machines (SVMs). Classification accuracy ranged from 87% to 97% for individual participants and from 62% to 96% when datasets were combined. In a subsequent study, the same dataset was used with an improved model architecture, resulting in a performance range of 90.1% to 99.3% [29].

In a health and safety-oriented study, Nath et al. [30] examined musculoskeletal risk by recognising activities such as lifting, loading, unloading, pushing, and pulling. Smartphones were affixed to the arm and waist of two workers, and each action was categorised into one of three ergonomic risk classes: no-risk (0), risky (1), and very risky (2). Using 12 extracted features and an SVM classifier, an accuracy of 90.2% was achieved, allowing the researchers to assess the duration of worker exposure to risky tasks.

Ryu et al. [31] studied the actions of ten masonry workers engaged in wall construction under laboratory conditions. Four categories of activities—applying plaster, carrying and placing bricks, arranging bricks, and removing excess plaster—were recorded via smartphones mounted on the active arm. Feature extraction was performed using window sizes ranging from 1 to 4 s. Among four evaluated ML algorithms, the highest classification accuracy of 88.1% was achieved using a 4-s window.

Lastly, Yang et al. [32] employed SVM-based classification to estimate physical workload levels in ironworking. Eight distinct activities—including placing, cutting, and lifting iron—were successfully recognised, with reported accuracies ranging between 94% and 99%.

These studies collectively affirm the growing relevance of sensor-based HAR in construction. However, most current efforts remain limited to classical ML methods. This study addresses a substantial research gap in applying DL architectures, such as LSTM and CNN, within real-world construction activity recognition.

## 3. Methods Used for Activity Recognition

Traditional ML techniques have historically played a central role in HAR. However, recent developments have increasingly favoured DL approaches, particularly CNNs and recurrent neural networks (RNNs), due to their superior ability to learn complex patterns directly from raw sensor data [33,34]. Unlike classical ML models, which typically require extensive domain expertise for manual feature engineering, DL models are capable of automatically extracting both spatial and temporal features, streamlining the development pipeline and enhancing classification performance.

CNNs, initially designed for image processing tasks, have demonstrated strong performance in HAR by transforming time-series sensor data into two-dimensional representations, enabling effective spatial pattern recognition. Moreover, one-dimensional CNNs (1D-CNNs) have been successfully employed to process raw inertial data directly, bypassing the need for manual transformation [35,36]. While CNNs are efficient at capturing local temporal correlations, they are inherently limited in modelling long-range dependencies, which are often critical in activity recognition tasks involving complex movement sequences [37].

In contrast, RNN architectures—particularly LSTM networks—are explicitly designed to model sequential dependencies, making them particularly suitable for analysing time-series data from wearable sensors. LSTM networks address the limitations of standard RNNs, such as the vanishing gradient problem, through gated memory units that retain relevant information across long time intervals. These architectures not only support real-time recognition by handling fine-grained temporal sequences (e.g., sampling rates of 50 Hz or higher) but also require less pre-processing than conventional ML methods, thereby offering a more streamlined and scalable solution for sensor-based HAR [14,38,39].

Given the temporal nature of the dataset employed in this study, comprising continuous motion signals from inertial sensors, a recurrent architecture was considered most appropriate for accurately modelling activity transitions and durations. Furthermore, the integration of LSTM layers facilitates dynamic temporal learning, thereby enabling high-resolution recognition across varied construction activities [40,41].

### 3.1. Deep Learning Architectures for Sensor-Based HAR

DL is a specialised subfield of ML that involves training ANNs with multiple hierarchical layers to automatically learn complex patterns from raw input data such as images, sensor signals, audio, or textual information. ANNs are composed of interconnected processing units, or neurons, which collaboratively learn to map inputs to outputs through iterative optimisation. The term “deep” refers to the depth of the network, defined by the number of hidden layers between the input and output nodes [42].

Among DL architectures, CNNs and RNNs are two of the most widely adopted models, particularly in fields that require spatial and temporal data analysis. CNNs are inspired by the structure of the visual cortex and are especially adept at identifying spatial hierarchies in image and signal data. They have been extensively employed for image recognition, video analysis, and, more recently, interpreting structured sensor inputs [43,44].

In contrast, RNNs are explicitly designed to handle sequential or time-dependent data, such as speech, handwriting, and time-series signals. Their architecture includes recurrent feedback loops and internal memory states, enabling them to retain contextual information over time and thereby model temporal dependencies [45,46,47].

Among the various RNN variants, LSTM networks and gated recurrent units (GRUs) have proven especially effective in preserving long-term dependencies and addressing the vanishing gradient problem during training [48,49,50]. These advanced recurrent architectures are particularly suited to applications such as HAR, where the model must capture both fine-grained and extended temporal dynamics. Recent studies consistently highlight their superior performance in time-series environments, with improvements in classification accuracy and robustness under varying sampling rates [51,52].

While CNNs remain prevalent in HAR research, particularly when visual or transformed time-series representations are used, RNN-based models—especially those employing LSTM and GRU mechanisms—are gaining increased traction in sensor-based HAR due to their inherent temporal modelling capabilities and flexibility in real-time applications.

### 3.2. Recurrent Neural Networks for Temporal Activity Modelling

RNNs are a class of ANNs distinguished by their recurrent connections, which allow the network to retain a form of internal memory. This architectural feature enables RNNs to exhibit temporal dynamic behaviour, rendering them highly suitable for processing sequential data. In contrast to feedforward neural networks, which assume input data samples are independent, RNNs exploit historical context to inform current predictions by maintaining a hidden state that evolves over time. The core computations within an RNN involve updating the hidden state and generating an output at each time step *t*, governed by the following equations:

(1) $$h^{\left(t\right)}=g_{h}\left(W_{i}x^{\left(t\right)}+W_{R}h^{\left(t-1\right)}+b_{h}\right)$$

(2) $${\hat{y}}^{\left(t\right)}=g_{y}\left(W_{y}h^{\left(t\right)}+b_{y}\right)$$

where

- $x^{\left(t\right)}$: input vector at time *t*;
- $h^{\left(t\right)}$: hidden state at time *t*;
- $h^{\left(t-1\right)}$: hidden state at time t−1;
- ${\hat{y}}^{\left(t\right)}$: predicted output at time *t*;
- $W_{i},W_{R},W_{y}$: weight matrices for input, recurrent, and output connections, respectively;
- $b_{h},b_{y}$: bias terms;
- $g_{h},g_{y}$: non-linear activation functions (commonly tanh or ReLU).

The principal strength of RNNs lies in their capacity to model temporal dependencies by preserving information across time steps. This sequential memory mechanism enables the network to recognise temporal patterns in time-series data, such as those generated via wearable sensors. Nevertheless, conventional RNNs are prone to the vanishing gradient problem, wherein gradients computed during backpropagation through time (BPTT) diminish exponentially. This significantly limits the model’s ability to capture long-range dependencies [53].

To address these limitations, advanced RNN architectures, such as LSTM networks [54] and GRUs [55], were developed. These variants introduce gating mechanisms that dynamically regulate the information flow across time steps, enabling the selective retention of memory and the effective propagation of gradients. Empirical studies have demonstrated the continued effectiveness of these models in time-series classification tasks, including HAR. For example, Modukuri et al. [2] and Tehrani et al. [3] reported notable improvements in classification accuracy and robustness when deploying LSTM and GRU networks on wearable sensor datasets.

As a result, LSTM and GRU-based architectures have become the standard choice for sequential learning tasks where robust temporal modelling is essential, offering enhanced performance over both traditional RNNs and classical ML techniques.

### 3.3. Long Short-Term Memory Networks for Long-Range Dependency Learning

**Figure 4:**
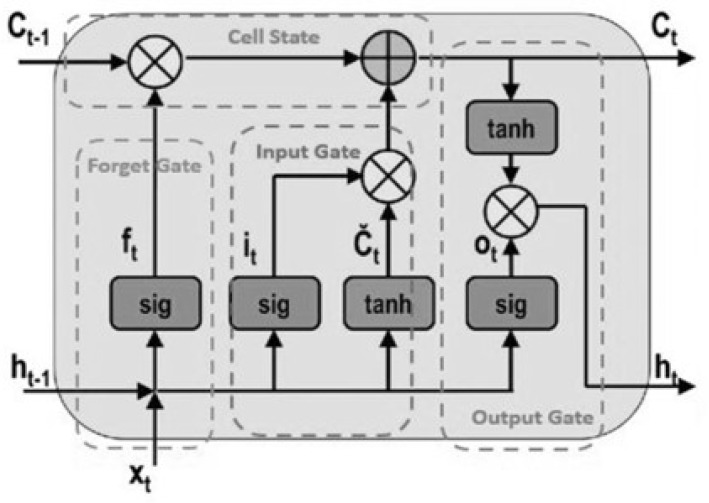
LSTM cell architecture [56,57].

LSTM networks, originally introduced by Hochreiter and Schmidhuber [54], were developed to address the vanishing gradient problem inherent in traditional RNNs. LSTM networks are specifically designed to capture and retain long-range temporal dependencies in sequential data by incorporating a memory cell mechanism that enables information to persist across extended time steps. The architecture of a standard LSTM cell is depicted in Figure 4.

Each LSTM unit comprises the following key components: the cell state ($C_{t}$), forget gate ($f_{t}$), input gate ($i_{t}$), and output gate ($o_{t}$). These elements work collectively to regulate the flow of information into, within, and out of the memory cell, thus enabling selective information retention and controlled updates.

- $C_{t}$: cell state (long-term memory);
- $f_{t}$: forget gate (controls what to remove from memory);
- $i_{t}$: input gate (controls what new information to store);
- $o_{t}$: output gate (controls what information to output);
- $h_{t}$: hidden state (short-term memory);
- σ: sigmoid activation function;
- tanh: hyperbolic tangent activation function.

The following set of equations governs the behaviour of an LSTM cell:

Forget gate—determines which information from the previous cell state to discard:

(3) $$\begin{array}{c} f_{t}=\sigma \left(W_{f}\left[h_{t-1},x_{t}\right]+b_{f}\right) \end{array}$$

Input gate—decides which new information to store in the cell state:

(4) $$i_{t}=\sigma (W_{i}\left[h_{t-1},x_{t}\right]+b_{i})$$

(5) $${\tilde{C}}_{t}=\tanh(W_{c}\left[h_{t-1},x_{t}\right]+b_{c})$$

Cell state update—updates the memory by combining retained and new information:

(6) $$\begin{array}{c} C_{t}=f_{t}\cdot C_{t-1}+i_{t}\cdot {\tilde{C}}_{t} \end{array}$$

Output gate—determines the output of the LSTM cell:

(7) $$\begin{array}{cc} o_{t} & =\sigma (W_{o}\left[h_{t-1},x_{t}\right]+b_{o}) \end{array}$$

(8) $$\begin{array}{cc} h_{t} & =o_{t}\cdot \tanh\left(C_{t}\right) \end{array}$$

In essence, the forget gate filters out irrelevant information, the input gate incorporates new relevant data into the memory cell, and the output gate determines what information to propagate to the next hidden state. Through this carefully regulated gating mechanism, LSTM networks are able to learn complex temporal relationships and preserve essential features over long sequences.

Due to these capabilities, LSTM networks have been extensively applied across a range of sequence learning tasks, including time-series forecasting, speech recognition, handwriting generation, and sensor-based HAR. Their resilience to gradient degradation and ability to manage variable-length input sequences make them a powerful tool in modelling dynamic human behaviours in both controlled and real-world environments.

### 3.4. Bidirectional Long Short-Term Memory Networks for Enhanced Temporal Context

Bidirectional long short-term memory (BiLSTM) networks extend the standard LSTM architecture by enabling the model to process input sequences in both forward and backward directions. This design builds upon the concept of bidirectional RNNs, initially introduced by Schuster and Paliwal [58]. A typical BiLSTM consists of two parallel LSTM layers: one processes the input sequence from past to future, while the other traverses it in reverse. The outputs from both directions are concatenated, enabling the network to make predictions based on the full temporal context.

**Figure 5:**
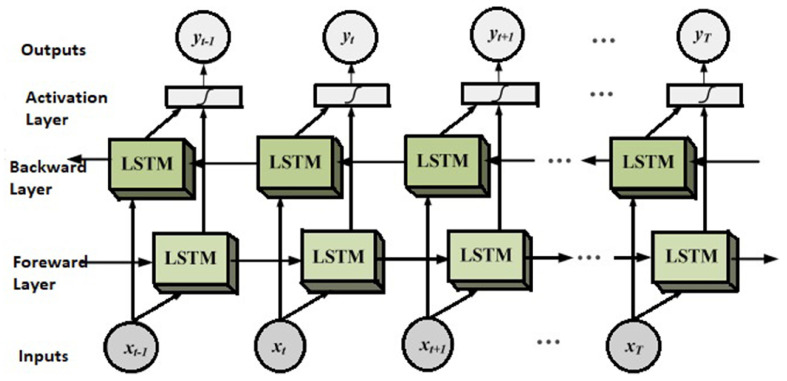
BiLSTM architecture [61].

This bidirectional structure allows BiLSTM networks to capture richer temporal dependencies, as they simultaneously consider both preceding and subsequent information in the sequence. Such capacity is particularly advantageous for time-series applications where future context can improve interpretability and predictive accuracy. The model architecture is illustrated in Figure 5.

Numerous empirical studies have demonstrated the superior performance of BiLSTM models over unidirectional LSTMs. For instance, Siami-Namini et al. [59] reported that BiLSTM networks achieved a 37.8% improvement in accuracy over LSTM in a benchmark time-series forecasting task. Likewise, in the domain of HAR, Ordóñez and Roggen [60] applied BiLSTM to the SKODA dataset and achieved significantly higher classification accuracy—reporting 88.4% for CNN and 95.8% for BiLSTM—highlighting the advantages of bidirectional temporal modelling in complex activity sequences.

By leveraging information from both temporal directions, BiLSTM networks offer enhanced sequence comprehension and contextual awareness. They are particularly well suited for sensor-based HAR applications where activities are temporally interdependent, such as in real-time recognition within dynamic and unpredictable environments.

## 4. Selected Activities and Sensor Data Collection

This study selected six distinct construction activities for HAR, representing a range of commonly performed site tasks with varied body postures and tool usage. The activities were as follows:

1. **Painting:** workers performed wall painting using a roller held with both hands, applying paint to a designated surface area.
2. **Plastering**: workers carried out wall plastering using a plastering trowel in their dominant (right) hand and a plasterer’s shovel in the other hand.
3. **Brick wall construction**: workers retrieved bricks from a nearby stack, applied mortar using a trowel, placed each brick on the wall, and tapped it with the trowel to ensure proper adhesion.
4. **Ceramic tile coating**: The task began with applying ceramic adhesive using a notched trowel. Workers then positioned a ceramic tile onto the prepared surface and tapped it several times using a plastic hammer to enhance adhesion.
5. **Laminate flooring**: Workers collected laminate planks from a stack and aligned them with previously installed flooring. Adhesion was improved by attaching a plastic tool to the new plank and tapping from both lateral sides.
6. **Wood panel installation**: workers lifted wooden panels from a nearby stack, aligned them on the wall, and secured them by hammering nails into the panel.

These activities were deliberately selected based on their movement and body positioning similarities to evaluate the system’s classification capability under complex and potentially confounding conditions. For example, both *painting* and *plastering* are performed while standing; *laminate flooring* and *ceramic tile coating* are conducted in a kneeling position, and *brick wall construction* and *wood panel installation* involve a combination of bending and upright postures.

By incorporating activity pairs with overlapping motion characteristics, the aim was to assess the robustness and precision of the DL-based recognition system in distinguishing between tasks with similar biomechanical patterns. This approach not only evaluates the model’s general accuracy but also its sensitivity in differentiating subtle variations in work-related motions under realistic construction site scenarios.

### 4.1. Sensors and Equipment Used for Data Collection

Notch motion sensors manufactured by Notch Interfaces Inc. were employed in this study to capture detailed human motion data. A total of 11 IMU sensors were used, each integrating an accelerometer, gyroscope, and magnetometer. These sensors were securely attached to designated parts of the workers’ bodies using adjustable rubber straps. Sensor management—including the initiation and termination of recordings—was conducted via the Notch Pioneer mobile application, operating on either a smartphone or a tablet.

**Figure 6:**
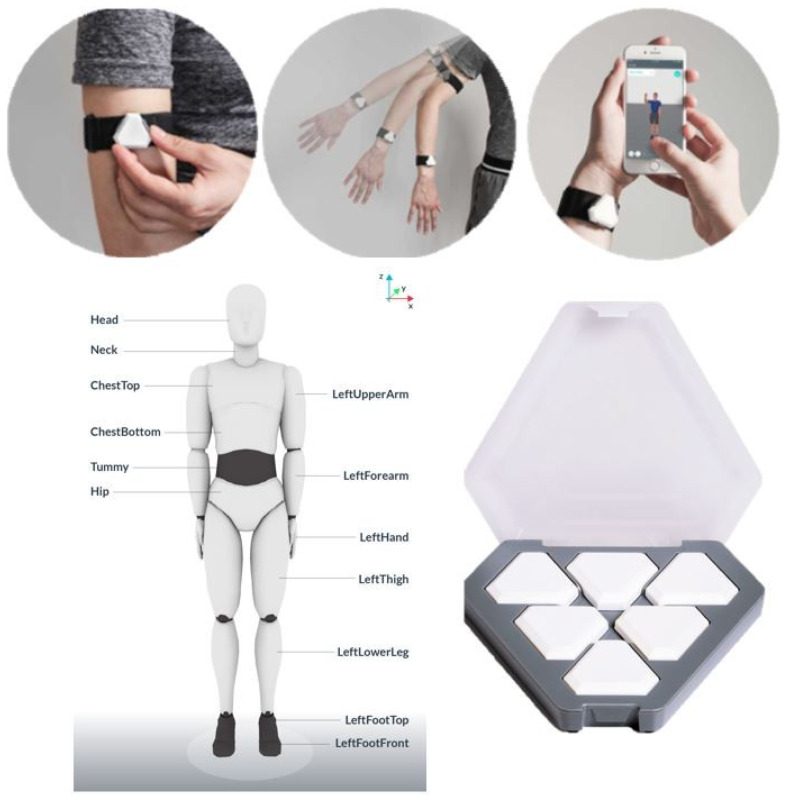
IMU sensors used for movement data collection [62].

The configuration and placement of the sensors are illustrated in Figure 6.

### 4.2. Data Collection Process

Data were acquired using 11 IMU sensors placed on five construction workers as they performed six distinct activities. The primary objective was to generate a robust dataset encompassing a range of movements and postures representative of actual construction tasks, thereby evaluating the classification performance of the DL model across different worker profiles.

Before the data collection was initiated, a literature review was conducted to establish appropriate sampling rates. Most previous HAR studies utilised sensor sampling frequencies between 50 Hz and 100 Hz. Based on these findings, a sampling rate of 50 Hz was selected for consistency with standard practices and to balance data resolution with storage and processing requirements.

**Figure 7:**
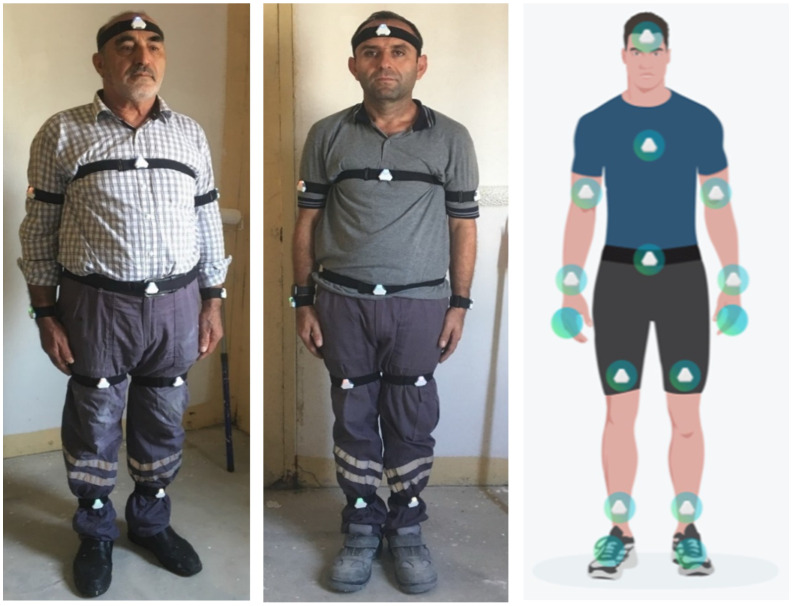
Placement of 11 IMU sensors on the construction workers.

The specific anatomical locations for the 11 sensors are depicted in Figure 7. These included both upper and lower limbs, torso, and head positions to ensure comprehensive coverage of movement patterns across the entire body.

Each sensor recorded four key motion parameters—linear acceleration, rotational velocity, rotational orientation, and positional displacement—along three axes (x, y, and z), amounting to 12 data points per sensor per time step. At a sampling rate of 50 Hz, each second of activity yielded 600 individual sensor readings (50 readings × 12 features). With 11 sensors deployed, this resulted in 6600 data points per second.

Data were collected over a sufficient duration for each activity to ensure statistical reliability, resulting in 6600 rows per activity segment. Throughout the study, a total of 53,169,600 data rows were recorded, representing approximately 8056 s of activity (roughly 134 min). This extensive dataset is the foundation for training and validating the DL models discussed in subsequent sections.

### 4.3. Ethical Considerations and Participant Recruitment

This study was approved by the Ethics Committee of Istanbul University-Cerrahpaşa, Institute of Science, located at Istanbul, Türkiye. All procedures adhered to the ethical standards outlined in the Declaration of Helsinki and the university’s research ethics guidelines.

Five adult construction workers (three male and two female), aged between 24 and 41 years, voluntarily participated in the study. Participants were required to have previous on-site construction experience and be physically capable of performing the selected tasks. Individuals with recent musculoskeletal injuries or any condition impairing mobility were excluded from participation to ensure both safety and the consistency of sensor data.

Each participant signed a written Voluntary Participation Consent Form prior to data collection. The form detailed the study’s purpose, procedures, potential risks, benefits, and the intended academic use of both sensor data and visual materials (e.g., photos and video recordings). It was explicitly stated that participation was entirely voluntary and that individuals could withdraw at any time without providing justification. In such cases, their data would not be used in the study.

To ensure privacy, participant names and identification numbers were not stored with the dataset. Sensor-collected data were anonymised before processing. Images captured during the study may appear in scientific publications or theses for academic purposes only, as agreed upon in the signed consent form.

## 5. Data Importing and Pre-Processing

This section outlines the steps to prepare the raw sensor data for input into the DL network. As the architecture employed in this study is based on an LSTM network, the collected data required structured pre-processing to enable effective training and validation. The workflow involved cleaning, segmentation, labelling, and data splitting.

### 5.1. Pre-Processing Pipeline

Following data acquisition, each sensor’s output was first synchronised and labelled according to the associated activity. This labelling step was essential for supervised learning and enabled the DL model to learn correlations between input patterns and activity classes.

During the manual inspection, it was observed that the initial 3–5 s of each activity recording typically consisted of preparatory motions—such as reaching for or adjusting tools—that were not representative of the core task. Given the sampling frequency of 50 Hz, this corresponds to approximately 150–250 rows per sensor. These rows were excluded to enhance classification performance by reducing noise and irrelevant transitions.

The refined dataset was then segmented using a sliding window approach. A window size of 6 s (i.e., 300 time steps) was applied, producing fixed-length sequences suitable for time-series input into the LSTM model. This segmentation procedure was applied uniformly across all experiments described in the following sections.

A separate test dataset was generated to evaluate model generalisability. A total of 18,300 rows—equivalent to 366 s of activity—were randomly withheld from each activity category, yielding 109,800 rows of test data. The remaining 384,300 rows were used for training. This results in a data split of approximately 71% training (274,500 rows) and 29% testing (109,800 rows). Table 1 presents the breakdown of test data across activity types.

An analysis of sensor contributions across different tasks revealed that hand and foot movements were particularly informative for activity recognition, highlighting these as the most critical sensor placements.

### 5.2. Experimental Configurations

To evaluate the performance of the LSTM model under different sensor modalities, three experimental configurations were designed:

- **Activity recognition with acceleration sensor data (ACC):** as most work-related motions involve upper limb activity, this experiment focused on acceleration data collected from the left and right hands.
- **Activity recognition with position sensor data (POS)**: position data from five key body parts—head, left and right hands, and left and right feet—were used to capture both upright and ground-based activities.
- **Combined acceleration and position data (ACCPOS)**: in this configuration, acceleration and position datasets were merged to assess whether data fusion could improve classification performance.

Distinct from many previous HAR studies that rely solely on acceleration or kinematic features, this work explores the contribution of positional data and their combination with acceleration signals. This multifaceted approach aims to enhance recognition accuracy, particularly under complex working conditions involving similar or overlapping movements.

## 6. Deep Learning Network and Training Results

### 6.1. Deep Learning Network Architecture

**Figure 8:**
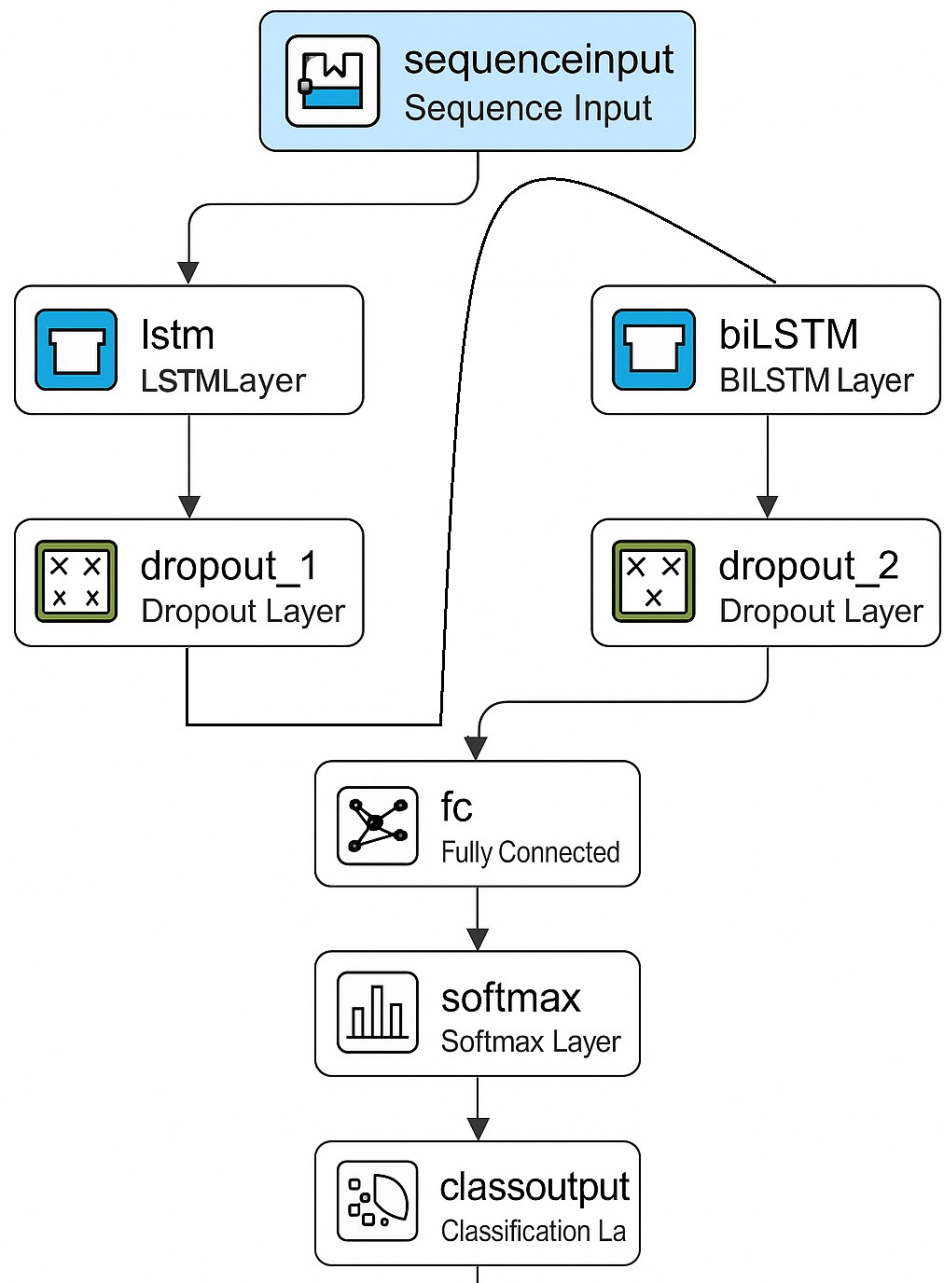
Schematic diagram of the final DL network architecture.

After the completion of data pre-processing, the resulting datasets were transferred to a DL network for training and evaluation. Initial experiments employed a simple architecture comprising a single LSTM layer and a dropout layer. However, this configuration achieved a maximum accuracy of only 86%. Several hyperparameters were optimised to improve performance, and the network architecture was expanded by introducing an additional BiLSTM layer. This led to a significant increase in classification accuracy. The final network architecture that yielded the best results is illustrated schematically in Figure 8.

### 6.2. Activity Recognition with Acceleration Sensor Data (ACC)

Only acceleration-related data from the left- and right-hand sensors were used in the first experimental configuration. This included readings from accelerometer, gyroscope, and magnetometer sensors along the x-, y-, and z-axes. A total of 274,500 rows (equivalent to 5490 s of activity) were used for training, while 109,800 rows (2196 s) of test data were held back for evaluation. Test data were not used during training to ensure unbiased performance assessment.

The optimised architecture incorporated one LSTM layer, one BiLSTM layer, and two dropout layers. Six different parameter sets were evaluated, as shown in Table 2. Each configuration was assessed based on accuracy metrics derived from training across three datasets: acceleration data only, position data only, and the combined dataset.

Parameter Set 3 yielded the highest training accuracy across all three datasets and was thus selected for use in subsequent evaluations. Using this configuration, the network achieved a training accuracy of 100% and a test accuracy of 98.1%.

Although combining acceleration (ACC) and positional (POS) data in the ACC+POS configuration was expected to yield consistently superior results due to its richer feature space, Table 2 shows that the training accuracy of ACC+POS does not always exceed that of ACC alone. This phenomenon can be attributed to a few factors. First, integrating multiple data modalities can introduce additional noise and redundancy if the features are not sufficiently decorrelated. In our dataset, certain motion patterns—particularly repetitive actions—may have been sufficiently captured via acceleration data alone, rendering the added positional inputs marginal or even disruptive during training. Second, feature fusion without dimensionality reduction or careful feature engineering may increase the risk of overfitting, especially in small-sample scenarios such as ours. Although dropout regularisation was applied, the additional complexity of fused data may not have been fully mitigated by the model’s structure or training strategy in all parameter configurations. Third, the positional data derived from IMUs is subject to drift and integration errors over time, which may have introduced subtle inconsistencies that influenced model convergence and generalisation during training. Despite these fluctuations in training accuracy, it is important to note that the test accuracy—as reported in Section 6—was highest for the ACC+POS configuration (99.59%), indicating better generalisation performance even when the training accuracy did not peak. This outcome reinforces the value of sensor fusion for real-world HAR systems, provided model complexity is well-managed and datasets are sufficiently large.

**Figure 9:**
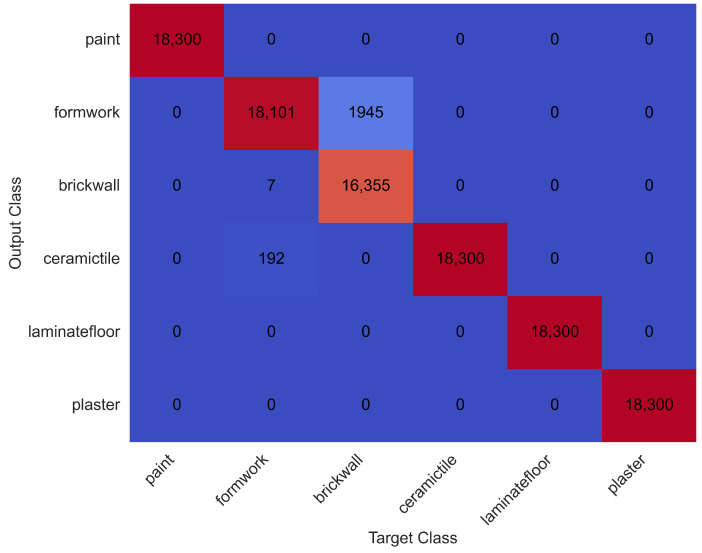
Confusion matrix for test data using acceleration sensor data.

The confusion matrix for test data, derived using Set 3 parameters, is presented in Figure 9.

**Figure 10:**
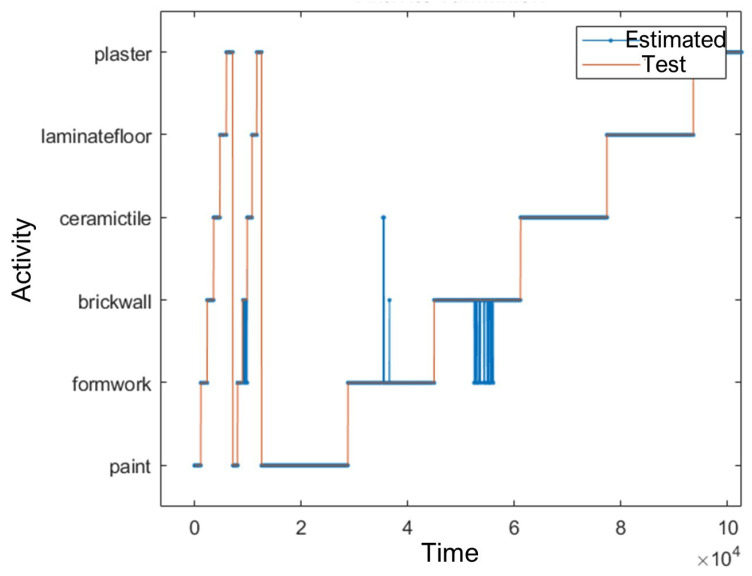
Time-series comparison of test data and predicted activities.

Among the six activity types, painting, plastering, ceramic tile coating, and laminate flooring were all classified with 100% accuracy. The brick wall building activity had the lowest classification accuracy, with a correct recognition rate of 89.4%. A time-series visualisation of test data versus predicted activity labels is shown in Figure 10.

A summary of performance metrics from the test set is provided in Table 3.

These results confirm the model’s high precision and generalisability in recognising complex human activities from hand-based motion sensor data. The performance gap observed in the brick wall activity suggests a higher level of intra-class variability in that task, possibly due to irregular movement sequences and transitions.

### 6.3. Activity Recognition with Position Sensor Data (POS)

This experiment used position data from five body parts—head, right hand, left hand, right foot, and left foot—for training and testing. A total of 274,500 rows (equivalent to 5490 s) of training data and 109,800 rows (2196 s) of test data were utilised. The same DL architecture and optimised parameter set (Set 3).

**Figure 11:**
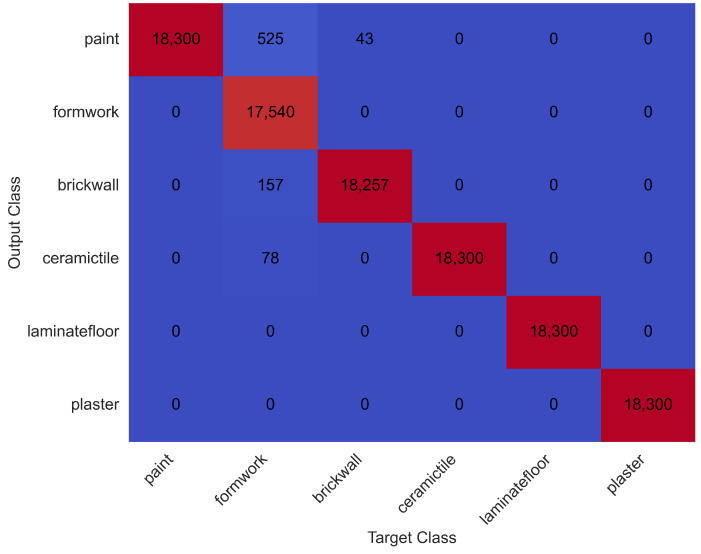
Confusion matrix for test data using position sensor data.

The model achieved a training accuracy of 98.5%. The resulting confusion matrix for test data is presented in Figure 11.

As in the acceleration-based study, four activities—painting, plastering, laminate flooring, and ceramic tile coating—were classified with 100% accuracy. The lowest classification accuracy was recorded for the wood coating activity (95.8%), with a small portion misclassified as either painting or brick wall building. Notably, the brick wall activity, which showed the lowest accuracy (89.4%) using acceleration data, improved substantially to 99.9% when using position data.

**Figure 12:**
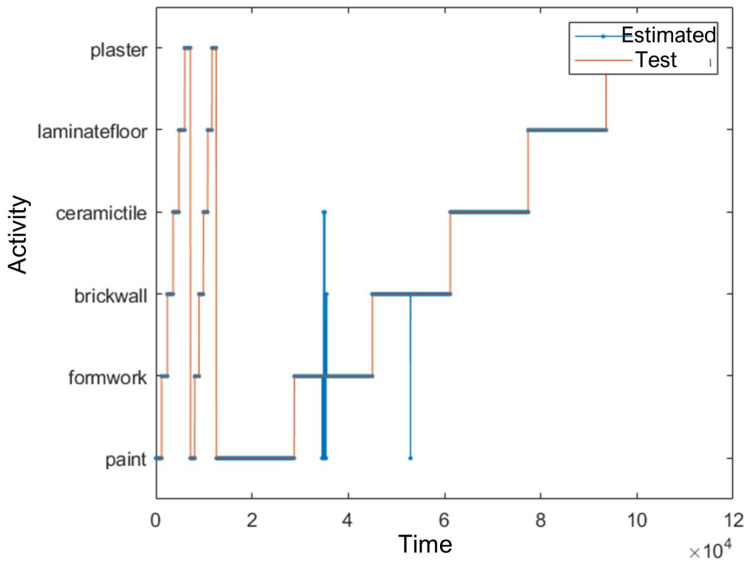
Time-series comparison of test data and predicted activities using position data.

Figure 12 shows the classification performance over time.

Recognition results are summarised in Table 4. Only 803 rows (16 s) were misclassified, representing a significant reduction in error compared to the 43 s of misclassification observed in the acceleration-based model.

### 6.4. Activity Recognition with Combined Data (Acceleration and Position, ACCPOS)

In the final experiment, acceleration data from the right- and left-hand sensors were combined with position data from the head, hands, and feet. The same number of training (274,500 rows) and test (109,800 rows) samples were used, and the network architecture and parameters remained unchanged from previous experiments.

**Figure 13:**
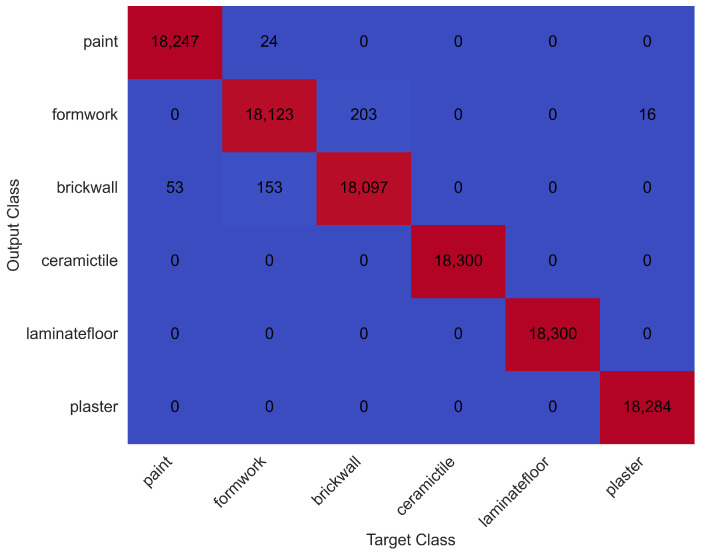
Confusion matrix for test data using combined acceleration and position data.

The combined dataset yielded the highest overall performance, with a training accuracy of 99.96%. Figure 13 provides the confusion matrix for test results.

Perfect classification (100%) was achieved for the ceramic tile coating and laminate flooring activities. The lowest accuracy rate was observed for brick wall buildings (98.9%). Overall, the results show a clear improvement in recognition accuracy for the previously problematic classes—brick wall and wood coating—which both achieved accuracies of approximately 99%.

**Figure 14:**
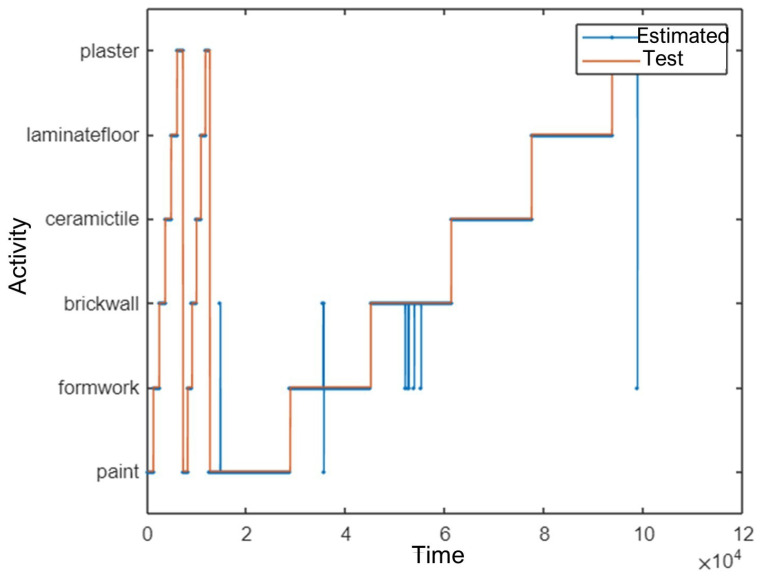
Test data versus predicted activity over time using combined sensor data.

A time-series comparison of predicted activity labels versus ground truth is illustrated in Figure 14.

A summary of classification performance using the combined dataset is shown in Table 5. Only 449 test data rows (9 s) were misclassified—the lowest error rate among all three experiments.

In conclusion, the combined use of acceleration and position data significantly enhanced the classification performance, particularly for tasks with subtle movement differences. The progression from 43 s of misclassified data (ACC) to 16 s (POS) and finally to only 9 s (ACCPOS) clearly demonstrates the added value of sensor fusion in HAR for construction scenarios.

### 6.5. Comparison of Dataset Performances and Activity Duration Prediction

The training parameters, recognition accuracy, and misclassification rates are summarised in Table 6 to compare the three experiments comprehensively. These results illustrate the clear performance improvement achieved when combining position data with acceleration signals.

**Figure 15:**
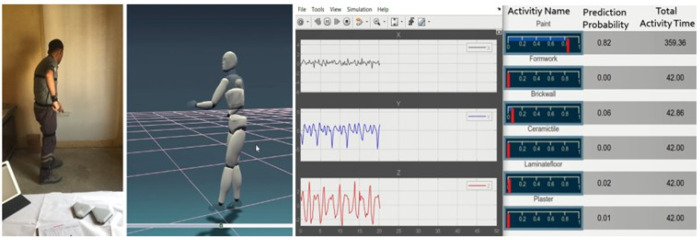
Screen captures of activity recognition and total activity time.

In addition to numerical evaluation, the trained network was applied to unlabelled test data to predict the worker’s activity and duration across a continuous recording. Figure 15 provides a visual output of this functionality, illustrating the model’s capability to support productivity and time management systems.

## 7. Discussion

The results of this study demonstrate the high efficacy of DL techniques—particularly LSTM and BiLSTM architectures—for recognising complex human activities in construction environments. Notably, the fusion of acceleration and position data yielded the best performance, achieving a test accuracy of 99.6% (see Table 7). This confirms the hypothesis that sensor fusion enhances recognition robustness, especially for activities with overlapping biomechanical patterns.

Compared to prior works, such as Akhavian and Behzadan [28], who reported accuracies ranging from 62% to 97% using smartphone-based ML classifiers, and Lim et al. [27], who achieved up to 94% for safety-related events, our method outperforms traditional ML models significantly. Furthermore, although recent DL-based HAR systems using public datasets have reported high accuracies—e.g., 98% using LSTM on Parkinson’s data [15], and 93–95% with CNN or hybrid models [60,61]—our results match or exceed these despite being based on more variable and noisy real-world construction data.

To clearly assess the generalisability and robustness of the proposed model, the dataset was partitioned into training and test subsets using a participant-based random splitting strategy. Specifically, while training data included samples from all five participants, the test dataset was randomly selected exclusively from the data of two participants. Importantly, these specific test samples were strictly excluded from the training set, although other distinct data from these two participants were included in the training process. This approach ensures an unbiased evaluation of the model’s performance while reflecting realistic conditions where partial familiarity with worker-specific patterns is expected.

In our study, all available sensor data were utilised due to the complex and dynamic nature of construction site activities, which involve frequent transitions and simultaneous limb movements. Although several studies propose sensor selection strategies to reduce system complexity—such as Borella et al. [63], who demonstrated that Shapley value-based sensor ranking can achieve comparable accuracy using a reduced subset—their work also showed that employing the full sensor setup yielded the highest classification performance (84.67%) in HAR experiments. Given the demanding nature of construction-related tasks, where subtle variations in motion may carry critical information, we opted to preserve the complete sensor configuration to ensure maximum recognition accuracy and system robustness.

This study also employed a 6-second sliding window with 50% overlap to segment sensor data for activity recognition. This configuration was selected to capture both short-term transitions and sustained movements with adequate temporal resolution. Prior studies support this choice: Mekruksavanich et al. [64] reported high accuracy using a 5-s window with 50% overlap in CNN-LSTM models, while Sun et al. [65] demonstrated that a fixed 5-s window achieved optimal results in HAR tasks. These findings corroborate the effectiveness of our windowing strategy in dynamic construction environments.

These findings underscore two key contributions:

- The inclusion of position data—often overlooked—markedly improves model discriminability.
- The BiLSTM architecture captures temporal dependencies more effectively than unidirectional LSTM, particularly for activities involving back-and-forth motion or transitions.

The practical implications are significant: such a system can be embedded in wearable safety gear to monitor workers’ task engagement in real time, assess productivity, and support site managers in resource allocation and planning. However, a number of limitations must be acknowledged, as detailed below.

## 8. Limitations

While the study demonstrates strong classification performance and practical relevance, several limitations must be acknowledged.

Firstly, the sample size is relatively small, involving only five participants. Although the selected individuals performed six representative construction activities and the dataset contains sufficient time-series samples for model training, the limited number of participants may not fully capture the diversity of body types, movement patterns, and task execution styles found across the broader construction workforce. This could potentially affect the model’s generalisability to larger or more heterogeneous worker populations.

Secondly, the data were collected under controlled conditions that, while reflective of real construction tasks, may not encompass all environmental and situational variances encountered on actual construction sites, such as weather changes, spatial constraints, or team-based interactions.

Moreover, although eleven IMU sensors were employed to ensure detailed activity capture, such a dense configuration may not be feasible in long-term site deployment due to comfort or practicality. Future research could explore the trade-off between sensor reduction and classification accuracy to develop lightweight, deployable HAR systems.

Lastly, the study focuses on six activities commonly observed in general construction, which, while representative, do not cover the full spectrum of roles or specialised tasks found in the industry. Expanding the activity set and diversifying participant demographics would be beneficial in future work to improve external validity.

These limitations suggest that further validation is needed under more diverse, large-scale, and field-deployed conditions to confirm the robustness and adaptability of the proposed framework.

## 9. Conclusions and Remarks

This study proposed a DL-based approach to automatically recognising construction worker activities using wearable sensor data. Six real-world construction tasks, with intentional overlap in posture and motion, were selected to evaluate model robustness in complex activity recognition scenarios. Data were collected from eleven IMU sensors worn by five workers, and three distinct datasets were created: acceleration-only, position-only, and a combined dataset.

The experiments demonstrated the following:

- Using only acceleration data yielded a test accuracy of 98.1%, highlighting the baseline capability of motion sensors.
- Position data significantly improved classification accuracy to 99.3%, reducing misclassification by 62%.
- Combining acceleration and position data further enhanced performance, achieving the highest test accuracy of 99.6% and reducing classification error by 79% relative to the acceleration-only model.

The results confirm that fusing multiple sensor modalities improves complex, subtle, or similar construction activity recognition. The study also demonstrated the feasibility of extracting total activity durations, enabling potential applications in real-time productivity tracking, progress assessment, and efficiency monitoring.

### 9.1. Contributions and Implications

This work contributes to the growing body of research on HAR in the construction domain as follows:

- Developing and validating a BiLSTM-based DL model trained on multimodal wearable sensor data.
- Demonstrating that position data—often overlooked in prior HAR studies—can substantially enhance recognition accuracy.
- Proposing a practical framework that can be adapted for future real-time, on-site monitoring applications.

### 9.2. Future Work

Several directions for future research are identified:

- Sensor placement sensitivity: future studies may explore the individual contribution of each sensor location to optimise performance while reducing sensor count.
- Sub-activity segmentation: complex tasks (e.g., bricklaying) can be divided into sub-activities (e.g., collecting, aligning, and applying mortar) to yield finer-grained insights.
- Scalability and deployment: transitioning this system to large-scale construction sites will require longer-lasting wireless sensors, real-time data processing capabilities, and strategies for addressing privacy and data protection concerns.

### 9.3. Final Remarks

The results of this study demonstrate that DL networks, especially those incorporating BiLSTM layers, offer a reliable and scalable method for recognising construction activities with high accuracy. Integrating positional data into traditional motion sensor frameworks significantly enhances model performance. These findings open new possibilities for intelligent construction site monitoring systems to improve efficiency, safety, and project management through automation and data-driven insights.

## Figures and Tables

**Table 1:** Test data details for different work activities.

Work Activity Type	Test Data (Rows)	Recording Duration (s)
Plastering	18,300	366
Painting	18,300	366
Wood Coating	18,300	366
Laminate Flooring	18,300	366
Brick Wall Building	18,300	366
Ceramic Tile Coating	18,300	366
Total	109,800	2196

**Table 2:** Training results for different parameter sets.

Parameter	Set 1	Set 2	Set 3	Set 4	Set 5	Set 6
Max Epochs	33	30	30	25	20	20
LSTM Layer Size	66	66	99	99	100	120
BiLSTM Layer Size	33	33	33	33	50	50
Dropout Layer 1	0.33	0.20	0.33	0.20	0.20	0.20
Dropout Layer 2	0.33	0.20	0.33	0.20	0.20	0.20
Train Accuracy (ACC)	99.18	98.52	99.97	99.19	98.96	99.41
Train Accuracy (POS)	98.38	97.53	99.51	98.57	98.92	99.43
Train Accuracy (ACC+POS)	98.66	98.31	99.44	98.27	98.72	99.70

**Table 3:** Recognition performance for acceleration data (ACC).

Metric	Value
Total Test Data	109,800 rows
Total Test Duration	2196 s
Accurately Recognised Data	107,656 rows
Accurate Recognition Duration	2153 s
Incorrectly Recognised Data	2144 rows
Incorrect Recognition Duration	43 s

**Table 4:** Recognition performance using position sensor data.

Metric	Value
Total Test Data	109,800 rows
Total Test Duration	2196 s
Accurately Recognised Data	108,997 rows
Accurate Recognition Duration	2180 s
Incorrectly Recognised Data	803 rows
Incorrect Recognition Duration	16 s

**Table 5:** Recognition performance using combined sensor data (ACCPOS).

Metric	Value
Total Test Data	109,800 rows
Total Test Duration	2196 s
Accurately Recognised Data	109,351 rows
Accurate Recognition Duration	2187 s
Incorrectly Recognised Data	449 rows
Incorrect Recognition Duration	9 s

**Table 6:** Comparison of recognition results across three datasets.

Metric	Acceleration Sensor	Position Sensor	Combined Sensors (ACC+POS)
Max Epochs	30	30	30
LSTM Layer Size	99	99	99
BiLSTM Layer Size	33	33	33
Dropout Layer 1	0.33	0.33	0.33
Dropout Layer 2	0.33	0.33	0.33
Training Accuracy (%)	100.00	98.48	99.96
Test Accuracy (%)	98.05	99.26	99.59
Misclassified Rows	2144	803	449

**Table 7:** Summary of recognition performance across sensor configurations.

Configuration	Train Accuracy (%)	Test Accuracy (%)	Misclassified Rows
Acceleration only (ACC)	100.00	98.05	2144
Position only (POS)	98.48	99.26	803
Acceleration + position (ACCPOS)	99.96	99.59	449

## Data Availability

Data will be made available on request.
